# Top‐down network analysis characterizes hidden termite–termite interactions

**DOI:** 10.1002/ece3.2313

**Published:** 2016-08-03

**Authors:** Colin Campbell, Laura Russo, Alessandra Marins, Og DeSouza, Karsten Schönrogge, David Mortensen, John Tooker, Réka Albert, Katriona Shea

**Affiliations:** ^1^ Department of BiologyPennsylvania State University208 Mueller LaboratoryUniversity ParkPennsylvania16802; ^2^ Department of PhysicsPennsylvania State University122 Davey LaboratoryUniversity ParkPennsylvania16802; ^3^ Department of PhysicsWashington CollegeChestertownMaryland21620; ^4^ Department of EntomologyCornell University3126 Comstock HallIthacaNew York14853; ^5^ Departamento de EntomologiaUniversidade Federal de ViçosaViçosaMG36570‐000Brazil; ^6^ Centre for Ecology & HydrologyNatural Environment Research CouncilMaclean BuildingBenson LaneCrowmarsh GiffordWallingfordOxfordshireOX10 8BBUK; ^7^ Department of Plant SciencesPennsylvania State University422 Agricultural Sciences and Industries BuildingUniversity ParkPennsylvania16802; ^8^ Department of EntomologyPennsylvania State University501 ASI BuildingUniversity ParkPennsylvania16802

**Keywords:** Antagonism, community interactions, host–parasitoid, inquilines, mound, mutualism, network structure, plant, pollinator, termite

## Abstract

The analysis of ecological networks is generally bottom‐up, where networks are established by observing interactions between individuals. Emergent network properties have been indicated to reflect the dominant mode of interactions in communities that might be mutualistic (e.g., pollination) or antagonistic (e.g., host–parasitoid communities). Many ecological communities, however, comprise species interactions that are difficult to observe directly. Here, we propose that a comparison of the emergent properties from detail‐rich reference communities with known modes of interaction can inform our understanding of detail‐sparse focal communities. With this top‐down approach, we consider patterns of coexistence between termite species that live as guests in mounds built by other host termite species as a case in point. Termite societies are extremely sensitive to perturbations, which precludes determining the nature of their interactions through direct observations. We perform a literature review to construct two networks representing termite mound cohabitation in a Brazilian savanna and in the tropical forest of Cameroon. We contrast the properties of these cohabitation networks with a total of 197 geographically diverse mutualistic plant–pollinator and antagonistic host–parasitoid networks. We analyze network properties for the networks, perform a principal components analysis (PCA), and compute the Mahalanobis distance of the termite networks to the cloud of mutualistic and antagonistic networks to assess the extent to which the termite networks overlap with the properties of the reference networks. Both termite networks overlap more closely with the mutualistic plant–pollinator communities than the antagonistic host–parasitoid communities, although the Brazilian community overlap with mutualistic communities is stronger. The analysis raises the hypothesis that termite–termite cohabitation networks may be overall mutualistic. More broadly, this work provides support for the argument that cryptic communities may be analyzed via comparison to well‐characterized communities.

## Introduction

Species interactions are a major driver of ecosystem structure and function. Well‐studied classes of species interactions include, for example, predator–prey and plant–pollinator interactions (see Ings et al. 2009 for a review). These species interactions are well studied in part due to their significant role in ecosystem stability and agricultural management. Another factor that contributes to the wealth of scientific effort that has been applied to these systems is the ease with which they may be observed: field observations often suffice to characterize predator–prey relationships and patterns of plant visitation by pollinators.

However, in many ecological communities, interactions are hidden, including microbial gut endosymbionts (Zindel et al. 2013), soil microfauna (Nottingham et al. 2013), gall‐parasitoid (Schönrogge and Crawley 2000), and interactions among species that co‐inhabit the nests of social insect societies such as ants (Thomas et al. 2005) or termites (Cristaldo et al. 2012). Characterizing these species interactions is challenging; yet, like their observable counterparts, determining the interaction types in these hidden communities is necessary for a complete description of ecological processes. Importantly, these interactions may be nontrophic, the study of which is necessary for a complete understanding of ecological function (Kéfi et al. 2012). Termites, for instance, are important ecosystem engineers (Jones et al. 1994) that have been shown to play a fundamentally important role in shaping ecosystem function, not only as significant bottlenecks to the flux of matter and energy (DeSouza et al. 2009) but also as hotspots of plant growth and animal productivity (Pringle et al. 2010; Bonachela et al. 2015) and diversity (Costa et al. 2009).

The development of analytic tools that may be used to characterize the interaction types in these hidden communities is therefore of significant interest. Network theory offers a promising framework for the development of such a tool. Indeed, network theory has already been used to inform our understanding of the structural and dynamical properties of a diverse body of ecological communities; notable examples include food webs (Dunne et al. 2002) and mutualistic communities of plants and their pollinators (Bascompte and Jordano 2007). In a network representation of an ecological community, species are represented as nodes, and their interactions are summarized with edges that connect the nodes. The topological properties of such an ecological network can yield significant insight into the represented community; indeed, it has been shown that the underlying structural properties of an ecological network are highly conserved and characteristic of community type (Bascompte and Jordano 2007; Thébault and Fontaine 2010). For instance, Thébault and Fontaine (2010) showed that mutualistic communities are inherently more nested than trophic communities. Despite individual interactions that counter the mutualistic or antagonistic nature of the entire network, such as the transmission of disease (McArt et al. 2014) or nectar‐robbing (Irwin et al. 2010) in pollinator networks, and herbivore attacks that enhance photosynthetic rates (Zhao and Chen 2012), the overall difference in structural properties is generally clear. Thus, by noting the interaction properties that are and are not common to communities in different ecological contexts, it is possible to gain insight into the drivers of community structure and, therefore, the mechanisms that shape the community's emergent ecosystem services.

This type of analysis is bottom‐up in the sense that networks are built by observing species–species interactions and recording the relevant information. At the most basic level, simply recording the existence of an interaction, such as one species consuming another, or one species of pollinator visiting a plant species, suffices. In this manner, detailed information about specific interactions is distilled into a network, which is in turn informative concerning the emergent properties of the community, such as its modularity or robustness (Pocock et al. 2012). However, in cases where we cannot directly observe the details of species–species interactions, can we reverse this process? That is, can network analysis of the emergent community serve as an effective top‐down analytical framework? We hypothesized that comparative analysis of the properties of known and hidden communities can inform, in an aggregate sense, our understanding of the nature of the constituent species–species interactions in the cryptic community.

In this report, we utilize this top‐down approach to consider the characteristics of termite communities cohabiting termite mounds, the so‐called “termite inquilines” sensu Araujo (1970). While the ubiquity of termite–termite associations, coupled with the stability of these associations during the lifespan of individual termites, suggests that negative interactions are largely avoided (Florencio et al. 2013), the difficulty in directly observing termite interactions makes this challenging to quantify. Termites are hidden in confined spaces and become highly stressed when exposed; direct observation of their interactions is not possible. Thus, the inter‐species interactions within termite mounds must be studied indirectly.

Indeed, even though several species of termites may cohabit in one mound (Araujo 1970), and some specific species‐level interactions may potentially be either mutualistic or antagonistic (Grassé 1986; Shellman‐Reeve 1997), the details of the interactions, and the community‐level properties that arise as a result, are relatively unstudied. For example, while the benefits to termite guests, such as buffered environment and access to nutrients, are easy to perceive (Silvestri 1903), the net gain of this association to host species is still obscure. Termite guests may reduce the space available inside the mound and feed off the host's storage reserves (Calaby 1956), but they may offset their parasitic use of space by inhabiting and maintaining unoccupied regions of the nest, building a hard shell around the host nest (Miura and Matsumoto 1997), and in some instances enhancing the defense of the mound against vertebrate (Redford 1984) and invertebrate (Higashi and Ito 1989) predators.

**Figure 1:**
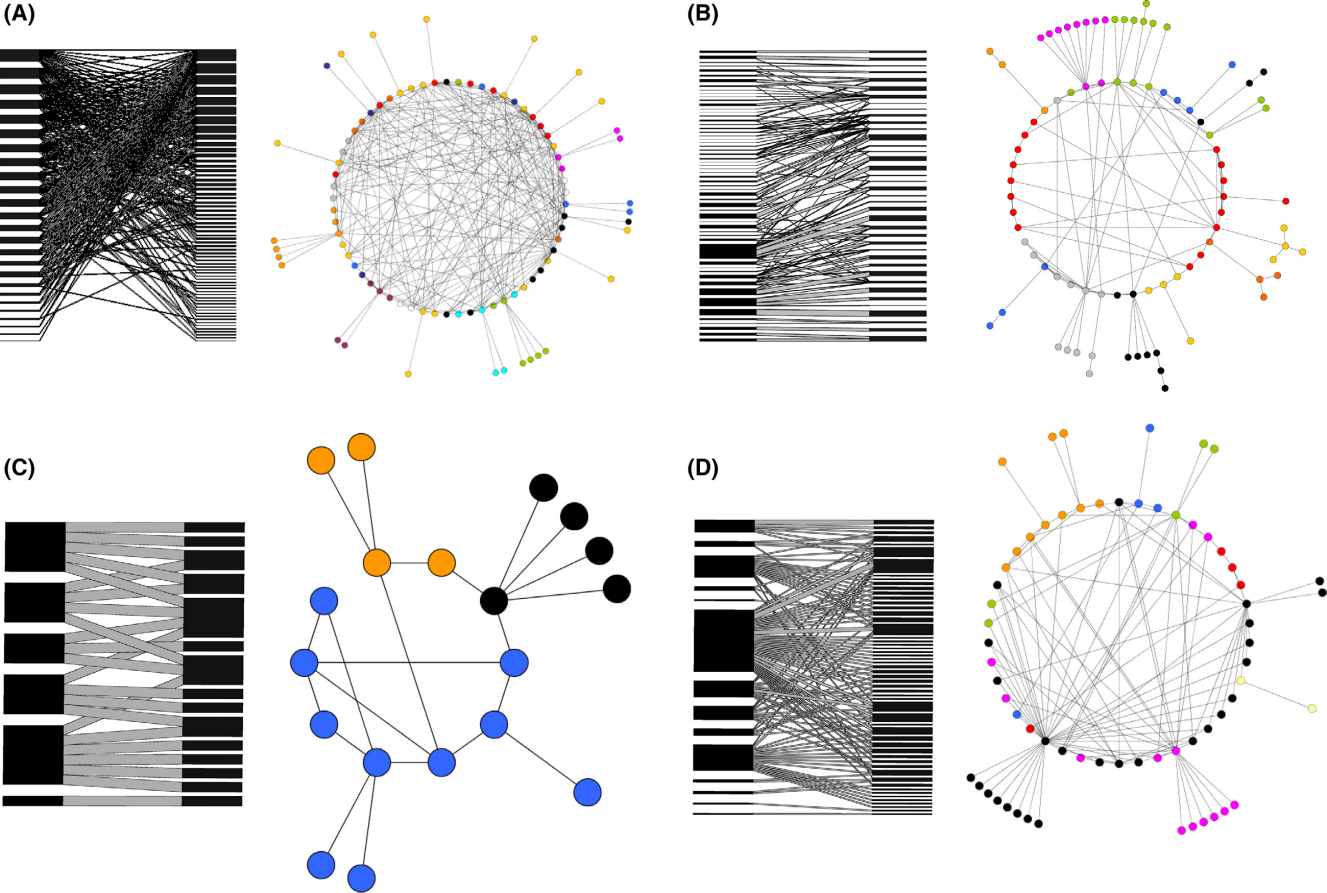
Visualization of (A) the plant–pollinator interaction network from (DeBarros 2010), (B) the host–parasitoid interaction network from (Memmott et al. 1994), (C) the Cameroon and (D) the Brazilian termite–termite networks. Each panel shows bipartite projections that emphasize nestedness (left) and the circular projections that show the compartmentalization structure (right), where colors indicate compartments and isolated compartments with ≤3 species are omitted.

In short, network interaction types potentially affect emergent network properties; in cryptic networks, where interactions are not directly observable, it may thus be possible to infer interaction types from higher‐level network properties. To assess the nature of within‐termite mound interactions, we survey the literature to generate termite–termite cohabitation networks, separately for the Afrotropical and Neotropical ecozones (specifically, the tropical forest of Cameroon and the Brazilian savanna). The networks comprise mound builder (host) termite species and the guest termite species found inside their mounds (see Methods). We compare these networks to 51 mutualistic (plant–pollinator) communities and 146 antagonistic (host–parasitoid) communities (see Methods) (Fig. 1). These bipartite communities occupy differing ends of the mutualism–antagonism spectrum and provide a broad basis for comparison to termite–termite communities. We consider standard network measures and discuss the properties of the termite communities in the context of the referenced community ensembles.

## Methods

In a network representation of a community, every species is represented as a node and the interactions between species are represented as links between those nodes (Bascompte and Jordano 2007). In a plant–pollinator network, links correspond to visitation of a plant species by a pollinator species; in a host–parasitoid network, links correspond to a parasitic relationship between the species; and in a termite network, links correspond to coexistence of a host and guest species within a nest/mound. The properties of these networks may be used to describe the structure of the communities they represent (Bascompte and Jordano 2007).

We consider a total of 197 empirical mutualistic and antagonistic communities, and thereby obtain a cloud of data that characterize both the limits and typical values of the properties of these community types. In the case of termite–termite communities, we build one network for a savanna‐like environment (Brazilian *cerrado*) and one for tropical forest (Cameroon forest); such data are extremely rare and difficult to obtain. Each network was built using several case studies where termite–termite cohabitation was recorded locally. Although some case studies were in different locations, all termite species occur throughout all locations within a given ecozone. We used 11 case studies for the savanna‐like environment (Brazilian *cerrado*) and three case studies for tropical forest (Cameroon forest) (Table S2).

### Analysis

The termite–termite communities are nearly bipartite (72 of 81 species act only as a host species or guest species). To facilitate comparisons with the bipartite reference communities, we consider bipartite projections of the termite–termite communities, where species that act as both hosts and guests are represented with both a host species node and a guest species node. Furthermore, the majority of the analysis presented below considers unweighted interaction matrices; the sole exception is the measurement of modularity, which is weighted by interaction frequencies, where available. Considering weighted interactions when evaluating modularity offers a more accurate summary of species interactions, and thereby offers greater fidelity in our comparison to coexistence between termite species.

While many network measures have been developed for the characterization of ecological networks (see e.g., Dormann et al. 2009), we here consider a representative sample of seven standard network measures (Table 1). *Species richness* (or network *size*, the number of nodes in the network representation of the community), *connectance* (the number of realized interactions relative to the number possible), and *asymmetry* (the distribution of the species between the two types, e.g., plants and pollinators) are basic network properties that provide a framework for higher‐level properties. While connectance is driven in large part by network size, plant–pollinator interactions networks have been shown to have higher connectance than some classes of antagonistic networks (Olesen et al. 2006), suggesting that plants and pollinators are relatively more generalized in their interactions. In addition, mutualistic plant–pollinator communities are more asymmetric than some antagonistic communities (Knops et al. 1999; Olesen and Jordano 2002); that is, there tend to be many more pollinators than plants in a given community, whereas antagonistic communities such as those comprising host–parasitoid interactions tend to have a more even distribution of species types.

**Table 1:** An overview of the structural measures used to characterize the networks considered in this report. Three basic measures are defined in terms of the number of species of each type (e.g., termite hosts and termite guests), *N* and *M*, and the number of observed interactions, *E*. These properties are preserved in a null model that randomizes the high‐level properties considered here (see Methods)

Measure	Description	Equation/References
**Controlled**		
Species richness	Total number of species	*N + M*
Connectance	Fraction of realized interactions	*E/(NM)*
Asymmetry	Balance between species types	*|N − M|/(N + M)*
**Randomized**		
Modularity	Degree of partitioning into weakly interconnected and tightly intraconnected groups	Newman and Girvan (2004)
Average clustering	Average density of local connections	Latapy et al. (2008)
Degree correlation	Tendency for species to interact with species with a similar number of interactions	Newman (2003)
Average redundancy	Average extent to which nodes are not necessary to maintain network connectivity	Latapy et al. (2008)

In addition to these three basic network properties, we consider four higher‐level properties. *Modularity* quantifies the extent to which a network is composed of tightly interacting modules (Newman and Girvan 2004). Ecological networks are generally modular (Ings et al. 2009); high values of modularity indicate that the community is characterized by modules such that many interactions exist within modules, but few exist between modules. High modularity, therefore, can correspond to a high degree of specialization (e.g., termite guests specializing with a particular host). Antagonistic networks are generally more modular than pollination networks (Olesen et al. 2007; Thébault and Fontaine 2010; Cagnolo et al. 2011; Wardhaugh et al. 2015); that is, they have a greater tendency to form modules of tightly interacting species.

We consider also *mean clustering*, which considers the average local density of interactions for a species (Latapy et al. 2008) and is a common metric for small‐worldness (Dormann et al. 2009); pollination networks have very high clustering (Olesen et al. 2006). Clustering may be considered a more local measure of specialization than modularity; for instance, high clustering may be a result of pollinator syndromes, where certain types of flowers attract groups of pollinators with complementary attributes.

Nestedness captures the tendency for a network to comprise well‐connected generalists and specialists that interact with subsets of the generalists; for example, open flowers tend to attract many pollinator species, including both generalists and specialists. Nestedness is quantified in many ways (see e.g., Almeida‐Neto et al. 2008; Staniczenko et al. 2013), and it has been shown that pollination networks are generally more nested than antagonistic networks (Thébault and Fontaine 2010; Cagnolo et al. 2011; Wardhaugh et al. 2015). As a proxy for nestedness, we here consider the *degree correlation*, or degree assortativity, which measures the tendency for nodes to be connected to nodes of similar degree (Newman 2003). In the context of bipartite ecological networks, high values suggest that generalists interact with generalists and specialists with specialists; low values suggest the opposite. As such, the degree correlation is related to the concept of nestedness; indeed, it has been shown that disassortative networks are nested and assortative networks are not (Jonhson et al. 2013).

Finally, we consider mean *redundancy*, which quantifies the extent to which nodes are not necessary to maintain connectivity in the network (Latapy et al. 2008). Low mean redundancy corresponds to a linear, or branchlike, community structure. Redundancy therefore provides a complementary view of network structure: networks with few generalists and many specialists may be nested according to some measures, but have low redundancy. Similarly, modular networks may have low redundancy depending on the structure of its modules.

Because high‐level network properties have been shown to depend in nontrivial ways upon basic network properties, especially the number of species (Dormann et al. 2009; Fortuna et al. 2010), we perform our analysis on both the set of all networks and a subset of the data restricted by overall size, such that all considered networks are of similar size to the termite interaction networks (specifically, the termite communities have 19 and 62 species; we restrict our analysis to networks in the range [10,70], i.e., approximately ±20% of the termite community range). In all cases, we consider a null model that generates a random bipartite network given the number of species in each class and the total number of interactions. Thus, the null model preserves connectance, network size, and network asymmetry. We report *Z*‐scores (i.e., the number of standard deviations that separates the termite communities' properties from the mean of the reference community properties) for 100 such randomizations. We consider both a property‐by‐property analysis of the termite communities compared to the reference communities and the termite communities' position in the complete cloud of reference network properties by way of a principal components analysis. Specifically in the latter case, we calculate the Mahalanobis distance, a generalized *Z*‐score (Mahalanobis 1936; Calenge et al. 2008), to assess the relative distance of the termite communities to the centroids in the distributions of mutualistic and antagonistic reference communities.

### Datasets

#### Plant–pollinator datasets

We analyzed a total of 51 plant–pollinator interaction webs; most were taken from the NCEAS Interaction Web DataBase and the dataset of Rezende et al. (2007). In addition, we considered (1) a taxonomically updated version of the dataset collected by Charles Robertson, comprising observations of insect species visiting flowering plant species in a tallgrass prairie ecosystem from 1884 to 1916 over an area of more than 225,000 hectares in central Illinois, USA (Robertson 1928; Tooker and Hanks 2000; Graham et al. 2012) and (2) a dataset comprising the interactions between 64 bee species (Apoidea) and 25 native perennial plant species in a common garden adjacent to both agricultural fields and forested lands (DeBarros 2010). We omitted the NCEAS web of Kevan (1970) from our analysis due to occasionally vague data entries.

Some datasets include quantitative information concerning interaction strength (e.g., visitation frequency). The edges in the network representations of these datasets were weighted according to these values, while edges in binary interactions networks received weights of 1 and 0 (present and absent, respectively). These values were used when calculating network modularity. The Robertson dataset does not have interaction strengths in the usual sense, but some interactions are noted as “frequent” or “abundant,” thereby giving three categories of interaction strength. Due to the atypically long‐term and broad nature of this study, we chose to focus on only the “abundant” interactions, reducing the network to 263 insect species visiting 215 plant species.

#### Host–parasitoid datasets

We considered a total of 146 host–parasitoid networks drawn from the literature; the studies range significantly in setting (see Table S1).

#### Termite–termite dataset

This binary dataset includes interactions between termite host species (mound builders) and other termite species found within the mounds (guests), independently for the tropical forest of Cameroon (19 species) and the Brazilian savanna (62 species) ecozones. Nine of 81 unique species act as both guests and hosts and are assigned unique “host” nodes and “guest” nodes in bipartite network projections, leading to a total of 90 unique nodes in the two networks. This dataset is based on 14 published and two unpublished studies (Table S2).

## Results

**Figure 2:**
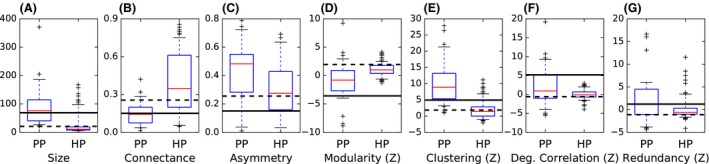
The properties of the mutualistic plant–pollinator (“PP”) and antagonistic host–parasitoid (“HP”) communities. The interquartile range is shown with a box; internal horizontal lines correspond to the median. Whiskers correspond to 5%, 95% percentiles, and outliers are marked with “+” symbols. The properties of the Cameroon termite–termite community are shown with a dashed horizontal line, and the properties of the Brazilian termite–termite community are shown with a solid line.

We find that the differentiation between mutualistic and antagonistic networks is in broad agreement with our expectations for both the full set of reference data (Fig. 2; see Methods) and the size‐restricted subset of data (Appendix S2).

The properties considered here highlight the similarities and differences between the termite communities (Fig. 2, horizontal lines). The Cameroon termite interaction network is smaller than the Brazilian network, has slightly greater connectance, and is somewhat more asymmetric. The Cameroon network is somewhat modular while the Brazilian network is not (*Z* = 1.9 vs. *Z* = −3.6), suggesting that guest species display greater specialization in host selection in the Cameroon network than in the Brazilian network, although this is mitigated to some extent by the fact that both communities are highly clustered (*Z* = 2.2 vs. *Z* = 4.9). The Brazilian network displays higher degree correlation (*Z* = −0.7 vs. *Z* = 5.2); this suggests that interacting pairs of species are more likely to either specialize with one another or to both coexist with other species in the Brazilian community than in the Cameroon community. The Brazilian community also displays greater redundancy (*Z* = −0.9 vs. *Z* = 1.2), indicating greater local overlap of species interactions, and to some extent greater local resilience to species loss.

The alignment of the termite communities with the mutualistic and antagonistic reference communities varies. The Brazilian community aligns more closely with the mutualistic communities for all measures except asymmetry, while the Cameroon community aligns more closely with the antagonistic communities for all measures except degree correlation, where it lies near the lower quartile for both groups of reference communities (Fig. 2).

We perform a principal components analysis of the data shown in Figure 2 and consider the Mahalanobis distance of the termite communities. We find that both termite communities are closer to the mutualistic plant–pollinator communities than the antagonistic host–parasitoid communities, although the difference is small in the case of the Cameroon community (*M* = 1.8 vs. *M* = 2.0 for the Cameroon community and *M* = 2.7 vs. *M* = 7.8 for the Brazilian community).

## Discussion

The interactions that form the basis of ecological communities shape their emergent structure (Bascompte and Jordano 2007; Thébault and Fontaine 2010). Here, we show that, as a result, it is possible to perform comparative top‐down analysis between communities with known and unknown interaction types. That is, when it is possible to generate network representations of communities based on simple information about species interactions, the analysis of the structure of the ensuing networks may allow us to understand the predominant characteristics of the constituent species interactions. In this report, we have performed such an analysis by comparing two independent networks that map the coexistence of termite species in termite mounds, to networks of well‐studied plant–pollinator (mutualistic) and host–parasitoid (antagonistic) interactions.

To obtain a holistic view of the structure of these networks, we consider several topological measures in addition to the basic measures of size (the number of species), connectance (the number of realized interactions relative to the number possible), and asymmetry (the relative number of each class of species). Of particular interest are clustering and modularity, which encapsulate differing mechanisms by which networks segregate into groups of tightly interacting species. For instance, the high overall clustering in the termite communities is related to connectance, insofar as both indicate that termite species are generally capable of co‐habitating with many other termite species. This is supported by the observation that termite inquilines are more affected by the attributes of termite mounds than by the host presence in the mounds (Marins et al. 2016). These properties may be related to the stability of these systems, as has been observed in other contexts (De Angelis 1975; Rozdilsky and Stone 2001; Dunne et al. 2002).

The other measures considered here, namely degree correlation and redundancy, respectively, characterize the similarity in patterns of interactions (based on the number of interactions per species) and the strength of network connectivity (see Methods; Table 1). These measures characterize many of the topological features of the networks considered here, and thereby facilitate a thorough comparison of their structures. The analysis raises the hypothesis that the Brazilian termite community aligns more closely with the mutualistic plant–pollinator communities than the antagonistic host–parasitoid communities; the signal is somewhat more ambiguous in the case of the Cameroon community.

**Figure 3:**
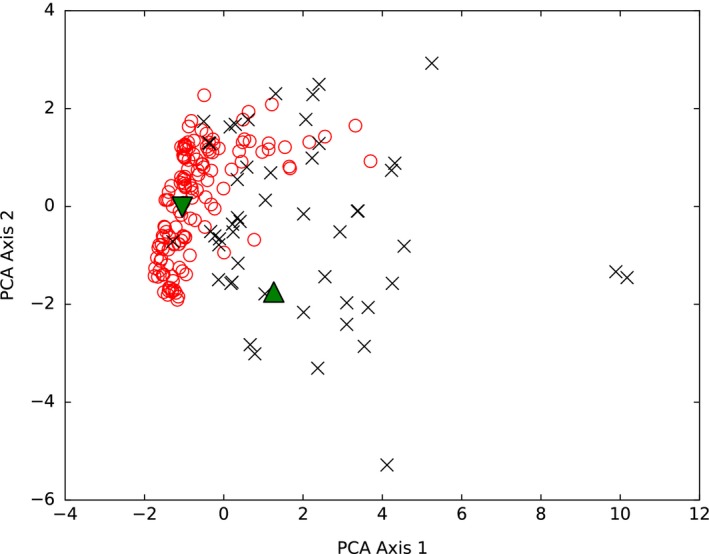
A principal component projection of community properties shown in Figure 2. Mutualistic plant–pollinator communities are shown with open red circles, and antagonistic host–parasitoid communities are shown with black crosses. The Cameroon community is shown with a downward green triangle, and the Brazilian community is shown with an upward green triangle. The component contributions for axis 1 are as follows: size—28%, clustering—23%, redundancy—18%, connectance—13%, modularity—12%, degree correlation—7%, asymmetry—0%; for axis 2 are as follows: asymmetry—50%, degree correlation—27%, connectance—13%, redundancy—7%, modularity—3%, and <1% for size and clustering.

We study these relationships in a more holistic sense by means of a principal components analysis (Fig. 3) coupled with a statistical analysis of the termite networks' property distribution relative to those of the reference communities. Both termite communities align more closely with the mutualistic reference communities than the antagonistic reference communities, though we note that the Cameroon community also overlaps with the host–parasitoid communities. However, the Mahalanobis distances (generalized *Z*‐score) are generally larger than 2, indicating that the properties of both termite communities diverge from the properties of the reference mutualistic communities. While a measure‐by‐measure comparison of community properties can be insightful, an aggregate approach (such as a principal components analysis coupled with appropriate statistical analyses) provides a more robust view of the manner in which these properties covary, and thereby facilitates greater understanding than univariate analysis.

As the ensembles of networks considered here occupy differing ranges of community sizes, connectances, and asymmetries (Fig. 2A–C), we repeated our analysis on a subset of the data that comprises communities with sizes near those of the termite communities; this did not qualitatively affect our results (Supporting information). Thus, while our results assign some level of mutualistic characteristics to both the termite communities, the relationship is stronger in the case of the Brazilian communities. However, several caveats apply to these findings. Because our reference termite networks are constructed from literature review and encompass species coexistence at a coarse (presence/absence) level, our analysis of these networks was necessarily restricted to measures that consider only binary, unweighted interactions. More detailed data that incorporated cohabitation frequency would enable the application of frequency‐dependent measures (see e.g., Dormann et al. 2009), which, in turn, would offer sharper insight into the topological structures of these networks. Moreover, factors necessarily omitted in this study clearly play a role in a holistic comparison of antagonistic and mutualistic interactions. Interaction intimacy, for instance, impacts measures such as modularity and nestedness (Guimarães et al. 2007; Fontaine et al. 2011). While the reference communities involve direct interactions such as pollination, some termite–termite interactions are indirect (e.g., ecosystem engineering); clearly the inclusion of more details of species interactions will serve to strengthen the predictions of a top‐down analysis as proposed here. Such an analysis presents an exciting avenue for further investigation.

Furthermore, the extent to which environmental and other contextual factors (as opposed to the mutualistic/antagonistic nature of species‐species interactions) shape the emergent community‐level topological properties analyzed here is unclear. For instance, termite–termite cohabitation is an inherently ongoing process, while plant–pollinator interactions are comparatively brief and occur on varying time scales (Russo et al. 2013). Such information, as it becomes available, must be integrated into a holistic comparison of network‐level properties of communities from different ecological contexts. In addition, the application of multiple measures is bound to provide apparent significance in some cases, and the practical import of such findings must be considered carefully; this is why the synthesis provided by our principal components analysis is so critical.

Nonetheless, the analysis presented here raises the hypothesis that within‐mound termite–termite interactions are, in aggregate, characteristically mutualistic. This hypothesis is supported with both univariate and multivariate approaches, although some ambiguity exists in that some termite–termite univariate parameters align more closely with the parameters of antagonistic communities than mutualistic communities. Furthermore, while a holistic analysis of the overlap of the community properties indicates that both the termite–termite communities align more strongly with the mutualistic reference communities, the relative strength of the overlap is not particularly high, especially in the case of the Cameroon community. Appropriate comparisons between bipartite and unipartite networks (e.g., food webs) will provide greater clarity to comparative top‐down analysis, especially as more empirical data become available.

Some ambiguity in this analysis is to be expected; many interactions are neither purely antagonistic nor purely mutualistic. Indeed, some interactions may be commensal (Florencio et al. 2013; Cristaldo et al. 2014). We described above that mutualistic pollinator webs can be affected by interactions that transmit disease or by nectar‐robbing species that exploit plants. Similarly, we know that some inquiline termite species fight members of the host species when confronted, despite adopting behaviors that lower their overall cost to their hosts (Florencio et al. 2013; Cristaldo et al. 2014). Other studies have provided evidence suggesting that some termite–termite interactions may be mutualistic; for example, *Termes* sp. build a protective hard shell around soft nests of *Hospitalitermes* sp. while profiting from the nests' materials as a source of nitrogen (Miura and Matsumoto 1997). Bronstein (1994) also pointed out that the mode of interaction between the same species might vary in time and can be dependent on a range of biotic or abiotic factors. Given that, it is possible that the observed overlap with both network types (mutualistic and antagonistic) is actually revealing that there is a blend of mutualistic and antagonistic interactions among termite cohabitants.

Our analysis suggests that the Cameroon termite–termite interactions might be less dominated by mutualistic interactions than Brazilian interactions. This may be at least partially attributable to the varying sizes of the termite–termite interaction networks, as some network properties can be difficult to detect for smaller networks (Ulrich and Gotelli 2007). Nonetheless, our analysis samples networks of varying size and is robust to a size‐restricted subcomparison (see Supporting information), suggesting that this observation may not be an artifact of the sizes of the termite–termite networks. A possible explanation is that phylogenetically, the Cameroon termite species are older (Bourguignon et al. 2015), which could suggest over evolutionary time a higher diversity of these interactions has arisen in Africa, following patterns in Lycaenid butterfly–ant interactions, where parasitic lifestyles are thought to have evolved from mutualistic ancestors (Pierce et al. 2002; Als et al. 2004; Thomas et al. 2005). In the future, the hypotheses raised here may be tested via direct observation of within‐mound termite–termite interactions (e.g., through the use of fiber‐optic cables). In addition, the development of termite interaction models that explore the relationships between emergent network properties and interactions types represents a promising area for theoretical work (see e.g., Pilosof et al. 2013; Russo et al. 2014).

This work demonstrates the utility of top‐down analysis of known and cryptic ecological communities, particularly where the interactions within ecological communities are difficult to observe, and the interplay between the emergent structure of species interactions and the functioning of ecological communities is unclear. The top‐down network theory framework we present here can yield insight into the positive and negative interactions within cryptic communities. This approach may also be applied to other hidden ecological systems, to characterize the nature of interactions and elucidate the relationships between (1) species interaction and (2) community structure and function. More generally, the analysis of emergent network properties may inform our understanding of the local structure of the network in nonecological contexts where the local structure is not known a priori.

## Conflict of Interest

None declared.

## Supporting information


**Appendix S1.** Compartmentalization of plant‐pollinator datasets.
**Appendix S2.** Analysis of size‐restricted data.
**Figure S1.** The properties of the mutualistic plant‐pollinator (“PP”) and antagonistic host‐parasitoid (“HP”) communities with sizes between 10 and 70 species.
**Figure S2.** A principal component projection of community properties show in Figure S1, considering only communities with between 10 and 70 species.
**Table S1.** We considered a total of 146 host‐parasitoid networks drawn from the study of Morris et al. (2014).
**Table S2.** The empirical termite‐termite interactions analyzed in this report.
