# Supplementary material for: Top‐down network analysis characterizes hidden termite–termite interactions
Source: Ecol Evol. 2016 Aug 3;6(17):6178–88. doi: 10.1002/ece3.2313 (PMC5016641; doi:10.1002/ece3.2313)
Supplement: Supplementary file 1 — Appendix S1. Compartmentalization of plant‐pollinator datasets. Appendix S2. Analysis of size‐restricted data. Figure S1. The properties of the mutualistic plant‐pollinator (“PP”) and antagonistic host‐parasitoid (“HP”) communities with sizes between 10 and 70 species. Figure S2. A principal component projection of community properties show in Figure S1, considering only communities with between 10 and 70 species. Table S1. We considered a total of 146 host‐parasitoid networks drawn from the study of Morris et al. (2014). Table S2. The empirical termite‐termite interactions analyzed in this report. [file ECE3-6-6178-s001.docx]

**Supporting Information**

**Appendix S1 - *Compartmentalization of plant-pollinator datasets***

We employ the compartment detection algorithm of Newman and Girvan (Newman & Girvan, 2004). While many approaches exist for the detection of compartments (see Fortunato, 2010 for a review), this algorithm offers an advantage in that it is possible to determine the compartmentalization of a community with weighted interactions. The primary measure of this algorithm is the modularity, *Q*, of a community. Low values of *Q* correspond to indistinct compartments (independent of the number of compartments detected), while communities with multiple, distinct compartments have values of *Q* near 1.

The frequency of an interaction (e.g. of a particular pollinator species visiting a particular plant species) not only represents the strength of the interaction (Bersier *et al.*, 2002), but presumably also reflects the abundance of species within the community.  That is, rare species will naturally interact with decreased frequency; species with especially high interaction frequencies serve as drivers of community compartmentalization.

To test this, we analyzed in detail the DeBarros (DeBarros, 2010) and Robertson (Robertson, 1928; Tooker & Hanks, 2000; Tooker *et al.*, 2002, 2006; Graham *et al.*, 2012) datasets. The Robertson high-frequency dataset consisted of those interactions rated as abundant (which reduces network size to 23% of the full community), while the DeBarros high-frequency dataset consisted of those interactions with >3% of the maximum observed frequency (which reduces network to 36% of the full community). In both cases, the high-frequency plant-pollinator communities are more modular (compartmentalized) than the corresponding full communities (0.00 in the full communities *cf.* .10 and .51 for the high-frequency DeBarros and Robertson communities, respectively). The DeBarros high-frequency subset is more connected ( .12 vs. .07) and nested (52 vs. 36) than the complete community, though no significant changes were observed in connectance for the larger Robertson community (.010 vs. .011), and nestedness decreased slightly (14 vs. 10).

Importantly, the high-frequency communities have more compartments, even though they are composed of fewer species. However, when the plant-pollinator data is partitioned in terms of insect taxonomic orders rather than interaction frequency, the modularity and distribution of compartments is largely unchanged relative to the full community. Because separating the data by taxonomic order does not have a similar effect in the plant-pollinator networks, the frequency of interaction likely plays a larger role than species taxonomy. In the case of plant-pollinator communities, this suggests that the abundant or highly generalist species have largely non-overlapping spheres of influence.

**Appendix S2 - *Analysis of size-restricted data***

To isolate the effects of community size, we repeat our analysis on a subset of networks with a total of between 10 and 80 species (i.e., those that fall approximately in the range occupied by the termite networks). The median values of the community ensembles retain their ordering compared to the full data set, and comparing the termite communities to the reference communities yields the same qualitative results (Fig. S1 cf. Fig. 2).

When considering the aggregate cloud of analytical measures and computing Mahalanobis distances for the termite communities, roughly the same patterns are observed as with the full set of data; the Cameroon network is roughly equidistant from both clouds of data (M = 2.13 and M = 2.15 for the antagonistic and mutualistic reference communities, respectively) while the Brazilian network is closer to the mutualistic cloud of data (M = 8.0 vs. M = 3.5).


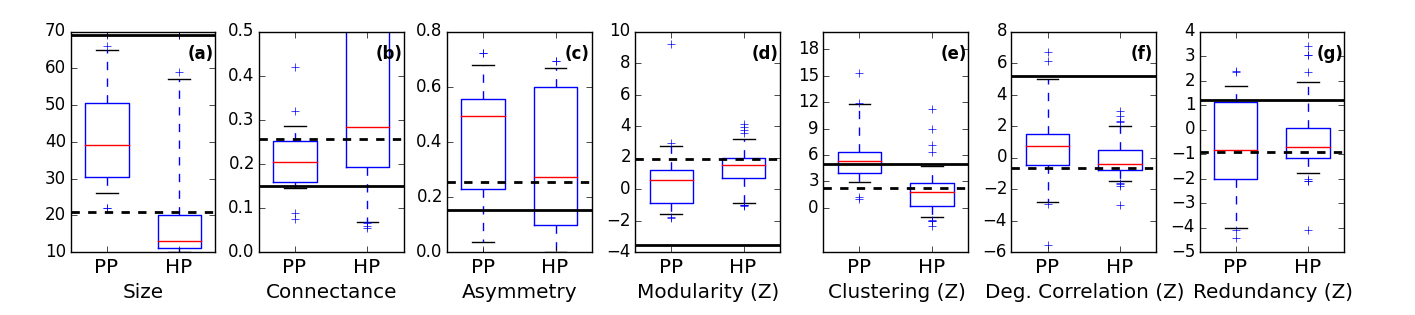


**Fig. S1.** The properties of the mutualistic plant-pollinator ("PP") and antagonistic host-parasitoid ("HP") communities with sizes between 10 and 70 species. The inter-quartile range is shown with a box; internal horizontal lines correspond to the median. Whiskers correspond to 5%, 95% percentiles, and outliers are marked with "+" symbols. The properties of the Cameroon termite-termite community are shown with a dashed horizontal line, and the properties of the Brazilian termite-termite community are shown with a solid line.


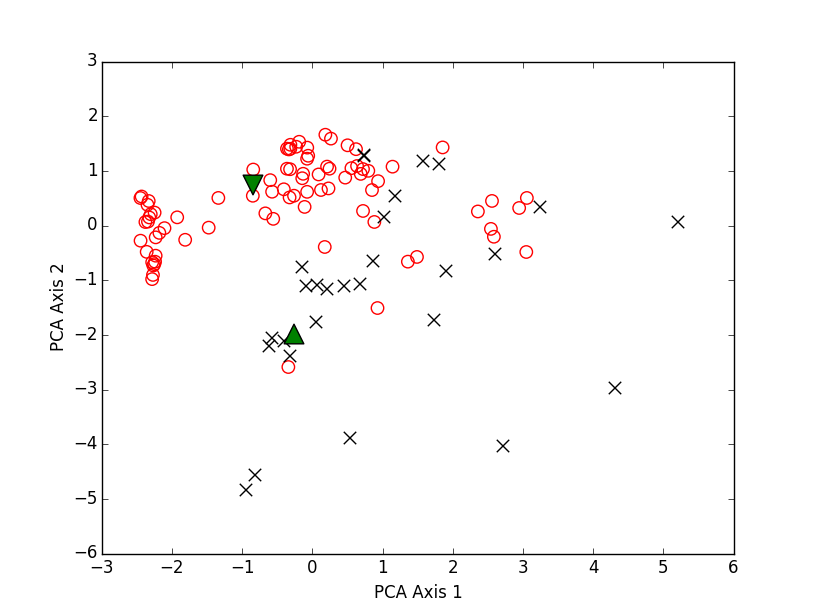


**Fig. S2.** A principal component projection of community properties show in Figure S1, considering only communities with between 10 and 70 species. Mutualistic plant-pollinator communities are shown with open red circles, and antagonistic host-parasitoid communities are shown with black crosses. The Cameroon community is shown with a downward green triangle, and the Brazilian community is shown with an upward green triangle. The component contributions for axis 1 are: connectance - 27.1%, size - 21.2%, redundancy - 19.9%, clustering - 13.7%, asymmetry - 13.5%, degree correlation - 3.5%, modularity - 1.1% and for axis 2 are: degree correlation - 30.2%, asymmetry - 24.9%, size - 19.3%, clustering - 17.6%, redundancy - 5.3%, modularity - 2.6%, connectance - <1%.

| **# Networks** | **Country** | **Host guild** | **Source** |
| --- | --- | --- | --- |
| 1 | Belgium | Aphids | (Alhmedi *et al.*, 2011) |
| 4 | USA | Leaf chewers | (Barbosa *et al.*, 2007) |
| 23 | Netherlands | Aphids | (Bukovinszky *et al.*, 2008) |
| 14 | Argentina | Leaf miners | (Cagnolo *et al.*, 2009) |
| 4 | UK | Leaf miners | (Carvalheiro *et al.*, 2010) |
| 1 | England | Leaf miners | (Clarke, 2000) |
| 5 | Germany | Trap nesters | (Gathmann *et al.*, 1994; Tscharntke *et al.*, 1998) |
| 9 | Germany | Trap nesters | (Tscharntke *et al.*, 1998) |
| 1 | USA | Leaf chewers | (Henneman & Memmott, 2001) |
| 24 | Finland | Leaf miners, Gallers | (Kaartinen & Roslin, 2011) |
| 18 | Indonesia | Trap nesters | (Klein *et al.*, 2006) |
| 1 | Belize | Leaf miners | (Lewis *et al.*, 2002) |
| 2 | UK | Leaf chewers, leaf miners | (Macfadyen *et al.*, 2009) |
| 1 | Costa Rica | Leaf miners | (Memmott *et al.*, 1994) |
| 4 | Australia | Trap nesters | R. Morris |
| 1 | UK | aphids | (Müller *et al.*, 1999) |
| 2 | Argentina | Aphids | (Omacini *et al.*, 2001) |
| 2 | Panama | Gallers | (Paniagua *et al.*, 2009) |
| 1 | Greenland | Leaf chewers | T. Roslin & G. Várkonyi |
| 1 | UK | Leaf miners | (Rott & Godfray, 2000) |
| 1 | France | Gallers | (Sinclair, 2012) |
| 26 | Ecuador | Trap nesters | (Tylianakis *et al.*, 2007) |

**Table S1.** We considered a total of 146 host-parasitoid networks drawn from the study of Morris et al. (2014). Data were provided by R. Morris with the permission of the original study authors, with the exception of the network of Sinclair (2012), which was provided by coauthor K. Schönrogge. These networks varied in size, taxonomic diversity, and geographical location. Some networks from Morris et al. (2014) were omitted due to low size interfering with assessment of the measures considered in this report. See Morris et al. (2014) for additional details on network properties and construction methodologies.

| **Termite Host** | **Termite Guest** | **Distribution** | **Source** |
| --- | --- | --- | --- |
| Apilitermes longiceps_H | Furculitermes winifredea | Cameroon | (Collins, 1980) |
| Apilitermes longiceps_H | Nasutitermes elegantulus | Cameroon | (Collins, 1980) |
| Apilitermes longiceps_H | Noditermes indoensis | Cameroon | (Collins, 1980) |
| Apilitermes longiceps_H | Orthotermes depressifrons | Cameroon | (Collins, 1980) |
| Cubitermes fungifaber | Coxotermes boukokoensis | Cameroon | (Collins, 1980) |
| Cubitermes fungifaber | Cubitermes_Heghi_G | Cameroon | (Collins, 1980) |
| Cubitermes fungifaber | Fastigitermes jucundus | Cameroon | (Collins, 1980) |
| Cubitermes fungifaber | Nasutitermes elegantulus | Cameroon | (Collins, 1980) |
| Cubitermes_Gaigei | Foraminitermes valens | Cameroon | (Collins, 1980) |
| Cubitermes_Heghi_H | Apilitermes longiceps_G | Cameroon | (Eggleton & Bignell, 1997) |
| Cubitermes_Heghi_H | Crenetermes albotarsalis | Cameroon | (Eggleton & Bignell, 1997) |
| Cubitermes_Heghi_H | Nasutitermes elegantulus | Cameroon | (Collins, 1980) |
| Cubitermes_Heghi_H | Noditermes indoensis | Cameroon | (Collins, 1980) |
| Cubitermes_Heghi_H | Orthotermes depressifrons | Cameroon | (Collins, 1980) |
| Cubitermes subarquatus | Apagotermes stolidus | Cameroon | (Dejean & Ruelle, 1995) |
| Cubitermes subarquatus | Cubitermes subcrenulatus | Cameroon | (Dejean & Ruelle, 1995) |
| Cubitermes subarquatus | Fastigitermes jucundus | Cameroon | (Dejean & Ruelle, 1995) |
| Cubitermes subarquatus | Furculitermes winifredea | Cameroon | (Dejean & Ruelle, 1995) |
| Cubitermes subarquatus | Orthotermes mansuetus | Cameroon | (Dejean & Ruelle, 1995) |
| Cubitermes subarquatus | Protermes prorepens | Cameroon | (Dejean & Ruelle, 1995) |
| Procubitermes arboricola | Furculitermes winifredea | Cameroon | (Collins, 1980) |
| Procubitermes arboricola | Nasutitermes diabolus | Cameroon | (Collins, 1980) |
| Procubitermes arboricola | Nasutitermes elegantulus | Cameroon | (Collins, 1980) |
| Embiratermes festivellus_H | Anoplotermes turricola | Brazilian savanna | (Araujo, 1970) |
| Embiratermes festivellus_H | Syntermes molestus_G | Brazilian savanna | (Araujo, 1958) |
| Procornitermes araujoi_H | Silvestritermes euamignathus_G | Brazilian savanna | (Araujo, 1958) |
| Procornitermes araujoi_H | Syntermes molestus_G | Brazilian savanna | (Araujo, 1958) |
| Silvestritermes euamignathus_H | Heterotermes longiceps | Brazilian savanna | (Araujo, 1958) |
| Silvestritermes euamignathus_H | Spinitermes brevicornutus | Brazilian savanna | (Araujo, 1958) |
| Cornitermes cumulans | Dihoplotermes inusitatus | Brazilian savanna | (Marins et. al. unpublished) |
| Cornitermes cumulans | Orthognathotermes_Heberi | Brazilian savanna | (Marins et. al. unpublished) |
| Cornitermes cumulans | Serritermes serrifer | Brazilian savanna | (Marins et. al. unpublished) |
| Cornitermes cumulans | Atlantitermes stercophilus | Brazilian savanna | (Marins et. al. unpublished) |
| Cornitermes cumulans | Crepititermes verruculosus | Brazilian savanna | (Marins et. al. unpublished) |
| Cornitermes cumulans | Curvitermes minor | Brazilian savanna | (Marins et. al. unpublished) |
| Cornitermes cumulans | Curvitermes odontognathus | Brazilian savanna | (Marins et. al. unpublished) |
| Cornitermes cumulans | Embiratermes festivellus_G | Brazilian savanna | (Marins et. al. unpublished) |
| Cornitermes cumulans | Heterotermes tenuis | Brazilian savanna | (Marins et. al. unpublished) |
| Cornitermes cumulans | Labiotermes orthocephalus | Brazilian savanna | (Marins et. al. unpublished) |
| Cornitermes cumulans | Neocapritermes parvus | Brazilian savanna | (Marins et. al. unpublished) |
| Cornitermes cumulans | Orthognathotermes mirin | Brazilian savanna | (Marins et. al. unpublished) |
| Cornitermes cumulans | Orthognathotermes okeyma | Brazilian savanna | (Marins et. al. unpublished) |
| Cornitermes cumulans | Silvestritermes euamignathus_G | Brazilian savanna | (Marins et. al. unpublished) |
| Cornitermes cumulans | Spinitermes robustus | Brazilian savanna | (Marins et. al. unpublished) |
| Cornitermes cumulans | Spinitermes trispinosus | Brazilian savanna | (Marins et. al. unpublished) |
| Cornitermes cumulans | Syntermes_Grandis_G | Brazilian savanna | (Marins et. al. unpublished) |
| Cornitermes cumulans | Velocitermes_Heteropterus_G | Brazilian savanna | (Marins et. al. unpublished) |
| Cornitermes silvestrii_H | Atlantitermes stercophilus | Brazilian savanna | (Cunha & Morais, 2010) |
| Cornitermes silvestrii_H | Embiratermes festivellus_G | Brazilian savanna | (Cunha & Morais, 2010) |
| Cornitermes silvestrii_H | Labiotermes brevilabius | Brazilian savanna | (Cunha & Morais, 2010) |
| Cornitermes silvestrii_H | Labiotermes laticephalus | Brazilian savanna | (Cunha & Morais, 2010) |
| Cornitermes silvestrii_H | Labiotermes longilabius | Brazilian savanna | (Cunha & Morais, 2010) |
| Cornitermes silvestrii_H | Neocapritermes araguaia | Brazilian savanna | (Cunha & Morais, 2010) |
| Cornitermes silvestrii_H | Rhynchotermes diphyes | Brazilian savanna | (Cunha & Morais, 2010) |
| Cornitermes silvestrii_H | Subulitermes microsoma | Brazilian savanna | (Cunha & Morais, 2010) |
| Cornitermes silvestrii_H | Syntermes nanus | Brazilian savanna | (Cunha & Morais, 2010) |
| Cornitermes silvestrii_H | Velocitermes_Heteropterus_G | Brazilian savanna | (Cunha & Morais, 2010) |
| Syntermes_Grandis_H | Cornitermes silvestrii_G | Brazilian savanna | (Cunha & Morais, 2010) |
| Syntermes_Grandis_H | Embiratermes festivellus_G | Brazilian savanna | (Cunha & Morais, 2010) |
| Syntermes_Grandis_H | Neocapritermes araguaia | Brazilian savanna | (Cunha & Morais, 2010) |
| Syntermes_Grandis_H | Subulitermes microsoma | Brazilian savanna | (Cunha & Morais, 2010) |
| Syntermes_Grandis_H | Velocitermes_Heteropterus_G | Brazilian savanna | (Cunha & Morais, 2010) |
| Constrictotermes cyphergaster | Inquilinitermes fur | Brazilian savanna | (Cunha *et al.*, 2003) |
| Constrictotermes cyphergaster | Inquilinitermes microcerus | Brazilian savanna | (Cunha *et al.*, 2003) |
| Cornitermes bequaerti | Cyrilliotermes strictinasus | Brazilian savanna | (Mathews, 1977) |
| Cornitermes bequaerti | Embiratermes festivellus_G | Brazilian savanna | (Mathews, 1977) |
| Cornitermes bequaerti | Genuotermes spinifer | Brazilian savanna | (Mathews, 1977) |
| Cornitermes bequaerti | Grigiotermes metoecus | Brazilian savanna | (Mathews, 1977) |
| Cornitermes bequaerti | Microcerotermes exiguus | Brazilian savanna | (Mathews, 1977) |
| Cornitermes bequaerti | Nasutitermes kemneri | Brazilian savanna | (Mathews, 1977) |
| Cornitermes bequaerti | Orthognathotermes aduncus | Brazilian savanna | (Mathews, 1977) |
| Cornitermes bequaerti | Paracurvitermes manni | Brazilian savanna | (Mathews, 1977) |
| Cornitermes bequaerti | Ruptitermes reconditus | Brazilian savanna | (Mathews, 1977) |
| Cornitermes bequaerti | Spinitermes trispinosus | Brazilian savanna | (Mathews, 1977) |
| Cornitermes bequaerti | Syntermes molestus_G | Brazilian savanna | (Mathews, 1977) |
| Cornitermes silvestrii_H | Angularitermes clypeatus | Brazilian savanna | (Mathews, 1977) |
| Cornitermes silvestrii_H | Curvitermes minor | Brazilian savanna | (Mathews, 1977) |
| Cornitermes silvestrii_H | Curvitermes odontognathus | Brazilian savanna | (Mathews, 1977) |
| Cornitermes silvestrii_H | Cyranotermes timuassu | Brazilian savanna | (Mathews, 1977) |
| Cornitermes silvestrii_H | Embiratermes festivellus_G | Brazilian savanna | (Mathews, 1977) |
| Cornitermes silvestrii_H | Grigiotermes metoecus | Brazilian savanna | (Mathews, 1977) |
| Cornitermes silvestrii_H | Heterotermes longiceps | Brazilian savanna | (Mathews, 1977) |
| Cornitermes silvestrii_H | Heterotermes tenuis | Brazilian savanna | (Mathews, 1977) |
| Cornitermes silvestrii_H | Labiotermes leptothrix | Brazilian savanna | (Mathews, 1977) |
| Cornitermes silvestrii_H | Microcerotermes exiguus | Brazilian savanna | (Mathews, 1977) |
| Cornitermes silvestrii_H | Orthognathotermes aduncus | Brazilian savanna | (Mathews, 1977) |
| Cornitermes silvestrii_H | Serritermes serrifer | Brazilian savanna | (Mathews, 1977) |
| Cornitermes silvestrii_H | Spinitermes nigrostomus | Brazilian savanna | (Mathews, 1977) |
| Cornitermes silvestrii_H | Spinitermes trispinosus | Brazilian savanna | (Mathews, 1977) |
| Cornitermes silvestrii_H | Termes medioculatus | Brazilian savanna | (Mathews, 1977) |
| Cornitermes silvestrii_H | Velocitermes_Heteropterus_G | Brazilian savanna | (Mathews, 1977) |
| Embiratermes neotenicus | Orthognathotermes aduncus | Brazilian savanna | (Mathews, 1977) |
| Labiotermes labralis | Cavitermes parvicavus | Brazilian savanna | (Mathews, 1977) |
| Syntermes molestus_H | Curvitermes minor | Brazilian savanna | (Mathews, 1977) |
| Syntermes molestus_H | Nasutitermes kemneri | Brazilian savanna | (Mathews, 1977) |
| Syntermes molestus_H | Velocitermes_Heteropterus_G | Brazilian savanna | (Mathews, 1977) |
| Cornitermes bequaerti | Cyrilliotermes angulariceps | Brazilian savanna | (Mathews, 1977) |
| Cornitermes bequaerti | Labiotermes leptothrix | Brazilian savanna | (Mathews, 1977) |
| Cornitermes bequaerti | Spinitermes nigrostomus | Brazilian savanna | (Mathews, 1977) |
| Cornitermes silvestrii_H | Anoplotermes turricola | Brazilian savanna | (Mathews, 1977) |
| Cornitermes silvestrii_H | Genuotermes spinifer | Brazilian savanna | (Mathews, 1977) |
| Cornitermes silvestrii_H | Labiotermes orthocephalus | Brazilian savanna | (Mathews, 1977) |
| Cornitermes silvestrii_H | Nasutitermes kemneri | Brazilian savanna | (Mathews, 1977) |
| Cornitermes silvestrii_H | Ruptitermes reconditus | Brazilian savanna | (Mathews, 1977) |
| Cornitermes silvestrii_H | Silvestritermes euamignathus_G | Brazilian savanna | (Mathews, 1977) |
| Silvestritermes euamignathus_H | Nasutitermes kemneri | Brazilian savanna | (Mathews, 1977) |
| Constrictotermes cyphergaster | Heterotermes longiceps | Brazilian savanna | (Florencio *et al.*, 2013) |
| Constrictotermes cyphergaster | Inquilinitermes microcerus | Brazilian savanna | (Florencio *et al.*, 2013) |
| Velocitermes_Heteropterus_H | Cyranotermes timuassu | Brazilian savanna | (Florencio *et al.*, 2013) |
| Velocitermes_Heteropterus_H | Heterotermes longiceps | Brazilian savanna | (Florencio *et al.*, 2013) |
| Velocitermes_Heteropterus_H | Heterotermes tenuis | Brazilian savanna | (Florencio *et al.*, 2013) |
| Velocitermes_Heteropterus_H | Labiotermes brevilabius | Brazilian savanna | (Florencio *et al.*, 2013) |
| Velocitermes_Heteropterus_H | Nasutitermes coxipoensis | Brazilian savanna | (Florencio *et al.*, 2013) |
| Velocitermes_Heteropterus_H | Procornitermes araujoi_G | Brazilian savanna | (Florencio *et al.*, 2013) |
| Velocitermes_Heteropterus_H | Silvestritermes euamignathus_G | Brazilian savanna | (Florencio *et al.*, 2013) |
| Velocitermes_Heteropterus_H | Spinitermes trispinosus | Brazilian savanna | (Florencio *et al.*, 2013) |
| Cornitermes cumulans | Velocitermes_Heteropterus_G | Brazilian savanna | (Redford, 1984) |
| Constrictotermes cavifrons | Inquilinitermes inquilinus | Brazilian savanna | (Emerson, 1938) |
| Cornitermes silvestrii_H | Angularitermes pinocchio | Brazilian savanna | (Cancello *et al.*, 1996) |
| Cornitermes silvestrii_H | Embiratermes festivellus_G | Brazilian savanna | (Cancello *et al.*, 1996) |
| Constrictotermes cyphergaster | Embiratermes festivellus_G | Brazilian savanna | (Costa unplublished data) |
| Constrictotermes cyphergaster | Inquilinitermes fur | Brazilian savanna | (Costa unplublished data) |
| Constrictotermes cyphergaster | Inquilinitermes microcerus | Brazilian savanna | (Costa unplublished data) |
| Constrictotermes cyphergaster | Nasutitermes kemneri | Brazilian savanna | (Costa unplublished data) |
| Constrictotermes cyphergaster | Parvitermes bacchanalis | Brazilian savanna | (Costa unplublished data) |
| Cornitermes bequaerti | Curvitermes odontognathus | Brazilian savanna | (Costa unplublished data) |
| Cornitermes bequaerti | Parvitermes bacchanalis | Brazilian savanna | (Costa unplublished data) |
| Cornitermes silvestrii_H | Cavitermes parmae | Brazilian savanna | (Costa unplublished data) |
| Cornitermes silvestrii_H | Curvitermes minor | Brazilian savanna | (Costa unplublished data) |
| Cornitermes silvestrii_H | Curvitermes odontognathus | Brazilian savanna | (Costa unplublished data) |
| Cornitermes silvestrii_H | Cyrilliotermes strictinasus | Brazilian savanna | (Costa unplublished data) |
| Cornitermes silvestrii_H | Embiratermes festivellus_G | Brazilian savanna | (Costa unplublished data) |
| Cornitermes silvestrii_H | Labiotermes emersoni | Brazilian savanna | (Costa unplublished data) |
| Cornitermes silvestrii_H | Nasutitermes kemneri | Brazilian savanna | (Costa unplublished data) |
| Cornitermes silvestrii_H | Nasutitermes longiarticulatus | Brazilian savanna | (Costa unplublished data) |
| Cornitermes silvestrii_H | Neocapritermes parvus | Brazilian savanna | (Costa unplublished data) |
| Cornitermes silvestrii_H | Spinitermes trispinosus | Brazilian savanna | (Costa unplublished data) |
| Cornitermes silvestrii_H | Velocitermes_Heteropterus_G | Brazilian savanna | (Costa unplublished data) |
| Cornitermes villosus | Cavitermes parmae | Brazilian savanna | (Costa unplublished data) |
| Cornitermes villosus | Curvitermes minor | Brazilian savanna | (Costa unplublished data) |
| Cornitermes villosus | Curvitermes odontognathus | Brazilian savanna | (Costa unplublished data) |
| Cornitermes villosus | Dihoplotermes inusitatus | Brazilian savanna | (Costa unplublished data) |
| Cornitermes villosus | Embiratermes festivellus_G | Brazilian savanna | (Costa unplublished data) |
| Cornitermes villosus | Genuotermes spinifer | Brazilian savanna | (Costa unplublished data) |
| Cornitermes villosus | Labiotermes emersoni | Brazilian savanna | (Costa unplublished data) |
| Cornitermes villosus | Nasutitermes kemneri | Brazilian savanna | (Costa unplublished data) |
| Cornitermes villosus | Serritermes serrifer | Brazilian savanna | (Costa unplublished data) |
| Cornitermes villosus | Spinitermes trispinosus | Brazilian savanna | (Costa unplublished data) |
| Silvestritermes euamignathus_H | Velocitermes_Heteropterus_G | Brazilian savanna | (Costa unplublished data) |
| Velocitermes_Heteropterus_H | Curvitermes odontognathus | Brazilian savanna | (Costa unplublished data) |
| Velocitermes_Heteropterus_H | Embiratermes festivellus_G | Brazilian savanna | (Costa unplublished data) |
| Velocitermes_Heteropterus_H | Neocapritermes parvus | Brazilian savanna | (Costa unplublished data) |
| Velocitermes_Heteropterus_H | Spinitermes trispinosus | Brazilian savanna | (Costa unplublished data) |

**Table S2.** The empirical termite-termite interactions analyzed in this report. The distribution of Afrotropical and Neotropical interactions follows (Constantino; Jones & Eggleton, 2011). "_H" and "_G" suffixes on species names correspond to species that may be both hosts and guests; the projection allowed for the calculation of nestedness. The studies referenced in this table met the following criteria: the host colony species differed from guest colony species, the host and guest species colony was identified to the specific level, and the host colony was active inside the mound. Specific names were updated according to Krishna (2013). Both unpublished studies (by co-authors: Costa and Marins) consisted of termite mounds sampled in search of its cohabitants (both builder and guest species).

Supporting Information References

Alhmedi, A., Haubruge, E., D’Hoedt, S. & Francis, F. (2011) Quantitative food webs of herbivore and related beneficial community in non-crop and crop habitats. *Biological Control*, **58**, 103–112.

Araujo, R.L. (1958) Contribuicao a biogeografia dos termitas de Minas Gerais Brasil (Insecta: Isoptera). *Aquivos do Instituto Biologico*, **25**, 219–236.

Araujo, R.L. (1970) *Termites of the Neotropical region*. *Biology of Termites* (ed. by K. Krishna) and F. Weessner), Academic Press.

Barbosa, P., Caldas, A. & Godfray, H.C.J. (2007) Comparative food web structure of larval macrolepidoptera and their parasitoids on two riparian tree species. *Ecological Research*, **22**, 756–766.

Bersier, L.-F., Banašek-Richter, C. & Cattin, M.-F. (2002) Quantitative descriptors of food-web matrices. *Ecology*, **83**, 2394–2407.

Bukovinszky, T., van Veen, F.J.F., Jongema, Y. & Dicke, M. (2008) Direct and Indirect Effects of Resource Quality on Food Web Structure. *Science*, **319**, 804–807.

Cagnolo, L., Valladares, G., Salvo, A., Cabido, M. & Zak, M. (2009) Habitat Fragmentation and Species Loss across Three Interacting Trophic Levels: Effects of Life-History and Food-Web Traits. *Conservation Biology*, **23**, 1167–1175.

Cancello, E.M., Brandao, D. & Amarante, S.T.P. (1996) Two new Angularitermes species (Isoptera, Termitidae,, Nasutitermitinae) from Brazil with a discussion of the cephalic microsculpture of the soldier. *Sociobiology*, **27**, 277–286.

Carvalheiro, L.G., Buckley, Y.M. & Memmott, J. (2010) Diet breadth influences how the impact of invasive plants is propagated through food webs. *Ecology*, **91**, 1063–1074.

Clarke, M. (2000) The impact of habitat fragmentation on community processes.

Collins, N.M. (1980) Inhabitation of epigeal termite (Isoptera) nests by secundary termites in Cameroun rain forest. *Sociobiology*, **5**, 47–54.

Constantino, R. On-line Termite Database.

Cunha, H., Costa, D.A., Santo, K.D., Silva, L.O. & Brandão, D. (2003) Relationship between Constrictotermes cyphergaster and inquiline termites in the Cerrado (Isoptera :Termitidae). *Sociobiology*, **42**, 761–770.

Cunha, H. & Morais, P. (2010) Relação Espécie-Área em Cupinzeiros de Pastagem, Goiânia-GO, Brasil. *EntomoBrasilis*, **3**, 60–63.

DeBarros, N. (2010) Floral resource provisioning for bees in Pennsylvania and the mid-Atlantic region.

Dejean, A. & Ruelle, J.E. (1995) Importance ofCubitermes termitaries as shelter for alien incipient termite societies. *Insectes Sociaux*, **42**, 129–136.

Eggleton, P. & Bignell, D.E. (1997) Secondary occupation of epigeal termite (Isoptera) mounds by other termites in the Mbalmayo forest Reserve, southern Cameroon, and its biological signiﬁcance. *Journal of African Zoology*, **111**, 489–498.

Emerson, A.E. (1938) Termite Nests--A Study of the Phylogeny of Behavior. *Ecological Monographs*, **8**, 247.

Florencio, D.F., Marins, A., Rosa, C.S., Cristaldo, P.F., Araújo, A.P.A., Silva, I.R. & DeSouza, O. (2013) Diet Segregation between Cohabiting Builder and Inquiline Termite Species. *PLoS ONE*, **8**, e66535.

Fortunato, S. (2010) Community detection in graphs. *Physics Reports*, **486**, 75–174.

Gathmann, A., Greiler, H.-J. & Tscharntke, T. (1994) Trap-nesting bees and wasps colonizing set-aside fields: succession and body size, management by cutting and sowing. *Oecologia*, **98**, 8–14.

Graham, E.E., Tooker, J.F. & Hanks, L.M. (2012) Floral Host Plants of Adult Beetles in Central Illinois: An Historical Perspective. *Annals of the Entomological Society of America*, **105**, 287–297.

Henneman, M.L. & Memmott, J. (2001) Infiltration of a Hawaiian Community by Introduced Biological Control Agents. *Science*, **293**, 1314–1316.

Jones, D.T. & Eggleton, P. (2011) *Global biogeography of termites: a compilation of sources*. *Biology of Termites: a modern synthesis* (ed. by D.E. Bignell), Y. Roisin), and N. Lo), pp. 477–498. Springer Science and Business Media.

Kaartinen, R. & Roslin, T. (2011) Shrinking by numbers: landscape context affects the species composition but not the quantitative structure of local food webs: Landscape effects on food webs. *Journal of Animal Ecology*, **80**, 622–631.

Klein, A.-M., Steffan-Dewenter, I. & Tscharntke, T. (2006) Rain forest promotes trophic interactions and diversity of trap-nesting Hymenoptera in adjacent agroforestry: Adjacent rain forest promote trophic interactions. *Journal of Animal Ecology*, **75**, 315–323.

Lewis, O.T., Memmott, J., Lasalle, J., Lyal, C.H.C., Whitefoord, C. & Godfray, H.C.J. (2002) Structure of a diverse tropical forest insect–parasitoid community. *Journal of Animal Ecology*, **71**, 855–873.

Macfadyen, S., Gibson, R., Polaszek, A., Morris, R.J., Craze, P.G., Planqué, R., Symondson, W.O.C. & Memmott, J. (2009) Do differences in food web structure between organic and conventional farms affect the ecosystem service of pest control? *Ecology Letters*, **12**, 229–238.

Mathews, A.G.A. (1977) *Studies on termites from the Mato Grosso State, Brazil*, Academia Brasileira de Ciências.

Memmott, J., Godfray, H.C.J. & Gauld, I.D. (1994) The Structure of a Tropical Host-Parasitoid Community. *Journal of Animal Ecology*, **63**, 521–540.

Morris, R.J., Gripenberg, S., Lewis, O.T. & Roslin, T. (2014) Antagonistic interaction networks are structured independently of latitude and host guild. *Ecology Letters*, **17**, 340–349.

Müller, C.B., Adriaanse, I.C.T., Belshaw, R. & Godfray, H.C.J. (1999) The structure of an aphid-parasitoid community. *Journal of Animal Ecology*, **68**, 346–370.

Newman, M.E.J. & Girvan, M. (2004) Finding and evaluating community structure in networks. *Physical Review E*, **69**, 026113.

Omacini, M., Chaneton, E.J., Ghersa, C.M. & Muller, C.B. (2001) Symbiotic fungal endophytes control insect host-parasite interaction webs. *Nature*, **409**, 78–81.

Paniagua, M.R., Medianero, E. & Lewis, O.T. (2009) Structure and vertical stratification of plant galler-parasitoid food webs in two tropical forests. *Ecological Entomology*, **34**, 310–320.

Redford, K.H. (1984) The Termitaria of Cornitermes cumulans (Isoptera, Termitidae) and Their Role in Determining a Potential Keystone Species. *Biotropica*, **16**, 112–119.

Robertson, C. (1928) *Flowers and insects: lists of visitors of four hundred and fifty-three flowers*, Carlinville, Ill.,.

Rott, A.S. & Godfray, H.C.J. (2000) The structure of a leafminer-parasitoid community. *Journal of Animal Ecology*, **69**, 274–289.

Sinclair, F.H. (2012) Community level consequences of adaptive management through Climate Matching: oak galls as a model system.

Tooker, J.F. & Hanks, L.M. (2000) Flowering Plant Hosts of Adult Hymenopteran Parasitoids of Central Illinois. *Annals of the Entomological Society of America*, **93**, 580–588.

Tooker, J.F., Hauser, M. & Hanks, L.M. (2006) Floral Host Plants of Syrphidae and Tachinidae (Diptera) of Central Illinois. *Annals of the Entomological Society of America*, **99**, 96–112.

Tooker, J.F., Reagel, P.F. & Hanks, L.M. (2002) Nectar Sources of Day-Flying Lepidoptera of Central Illinois. *Annals of the Entomological Society of America*, **95**, 84–96.

Tscharntke, T., Gathmann, A. & Steffan-Dewenter, I. (1998) Bioindication using trap-nesting bees and wasps and their natural enemies: community structure and interactions. *Journal of Applied Ecology*, **35**, 708–719.

Tylianakis, J.M., Tscharntke, T. & Lewis, O.T. (2007) Habitat modification alters the structure of tropical host–parasitoid food webs. *Nature*, **445**, 202–205.
